# LC-MS/MS Method Development and Validation for Clinical Pharmacokinetics and Therapeutic Drug Monitoring of Potassium-Competitive Acid Blocker Vonoprazan-Based Triple Therapy for *H. pylori* in Human Plasma

**DOI:** 10.3390/ph18101509

**Published:** 2025-10-08

**Authors:** Naser F. Al-Tannak, Hani M. Hafez, Ahmed Hemdan, Abdullah Aldasem, Ibrahim Khadra

**Affiliations:** 1 Department of Pharmaceutical Chemistry, Faculty of Pharmacy, Kuwait University, Kuwait City P.O. Box 46300, Kuwait; ahmad.hemdan@ku.edu.kw; 2 Pharmacy College, Al-Esraa University, Baghdad 10069, Iraq; hanyhafez82@gmail.com; 3 Strathclyde Institute of Pharmacy and Biomedical Sciences, University of Strathclyde, 161, Cathedral Street, Glasgow G4 0RE 1, UK; aldasem85@gmail.com (A.A.); ibrahim.khadra@strath.ac.uk (I.K.)

**Keywords:** LC-MS/MS, *H. pylori* infection, amoxicillin, vonoprazan, clarithromycin, green chemistry

## Abstract

**Background:** A novel triple therapy regimen for *Helicobacter pylori* eradication, recently approved by the U.S. FDA, comprises vonoprazan (VPN), a potassium-competitive acid blocker, in combination with amoxicillin (AMX) and clarithromycin (CMN). This study presents the development and full validation of a rapid, selective, and sensitive LC-MS/MS method for the simultaneous quantification of these three drugs in spiked human plasma. **Methods:** Sample preparation was performed using a simple and efficient liquid–liquid extraction (LLE) technique. Chromatographic separation was achieved within 5 min using a Phenomenex Kinetex C18 column (100 × 4.6 mm, 2.6 µm) and a gradient elution system consisting of 0.1% formic acid in water and acetonitrile. Moreover, diazepam was used as an internal standard. The mass spectrometric detection was conducted in multiple reaction monitoring (MRM) mode using positive electrospray ionization. **Results:** The method exhibited excellent linearity over the investigated concentration ranges (2, 5, 10, 20, 50, and 100 ng/mL for amoxicillin and clarithromycin and 5, 10, 20, 30, 50, and 100 ng/mL for vonoprazan). Intra- and inter-day precision and accuracy values met FDA bioanalytical method validation guidelines, with relative standard deviations and relative errors below 15%. Mean absolute recoveries were above 93% for all analytes. **Conclusions:** The developed method was fully validated, rapid, selective, and sensitive LC-MS/MS and was assessed using the AGREE tool as a greenness assessment approach, confirming its environmental friendliness and alignment with green analytical chemistry principles.

## 1. Introduction

Gastric ulcers are widespread gastrointestinal disorders characterized by lesions in the lining of the stomach. These ulcers result from the destructive effects of gastric acid and pepsin on the mucosal barrier. Several factors contribute to the development of gastric ulcers, with *Helicobacter pylori* (*H. pylori*) infection being a primary cause. Other contributing factors include the prolonged use of non-steroidal anti-inflammatory drugs (NSAIDs), excessive alcohol consumption, cigarette smoking, and chronic psychological stress. Although gastric ulcers are non-communicable, *H. pylori* infection, which plays a major role in ulcer formation, can be transmitted from person to person through direct oral contact or indirectly via contaminated water and food sources [1]. Successful eradication of *H. pylori* is essential in preventing ulcer recurrence and lowering the risk of complications such as gastrointestinal bleeding, perforation, and the potential development of gastric malignancies [2,3].

Standard treatment for *H. pylori* infection commonly involves triple therapy, which typically combines a proton pump inhibitor (PPI) with two antibiotics, such as amoxicillin and clarithromycin or metronidazole [4,5,6]. PPIs like omeprazole and lansoprazole work by suppressing gastric acid secretion, thereby creating a favorable environment for antibiotics to act more effectively against *H. pylori*. Despite its long-standing clinical utility, the traditional triple therapy regimen has encountered several limitations. These include the increasing prevalence of antibiotic resistance, particularly to clarithromycin and metronidazole, which significantly reduces eradication success rates. Additionally, variability in patient response due to genetic differences in PPI metabolism has been reported, further contributing to treatment failures [7]. These challenges have prompted researchers and clinicians to explore alternative strategies that provide more consistent therapeutic outcomes and improved efficacy.

One of the most promising recent developments in this area is the introduction of potassium-competitive acid blockers (P-CABs), which represent a new class of acid-suppressing agents. Unlike PPIs, P-CABs do not require acid activation and provide rapid, reversible, and prolonged inhibition of the gastric H^+^/K^+^-ATPase enzyme. Among these, vonoprazan has gained particular attention due to its superior pharmacological profile, which includes a faster onset of action, greater stability in acidic environments, and more sustained acid suppression compared to conventional PPIs [8,9,10]. These properties not only enhance patient compliance but also increase the efficacy of the accompanying antibiotics by maintaining an optimal gastric pH for a longer duration.

In response to these advantages, a new vonoprazan-based triple therapy was introduced and approved by the U.S. Food and Drug Administration (FDA) in 2022. Marketed under the trade name Voquezna Triple Pak^®^, this fixed-dose combination includes vonoprazan fumarate (20 mg), amoxicillin (500 mg), and clarithromycin (500 mg) [11]. The incorporation of vonoprazan into the therapy offers a more potent and consistent acid-suppressive effect, thereby overcoming the limitations associated with PPI-based regimens. Vonoprazan’s reversible inhibition of the gastric proton pump provides greater control over intragastric pH levels, enhancing the antimicrobial action of both amoxicillin and clarithromycin [12,13,14].

Several analytical techniques have been developed to support therapeutic monitoring and pharmacokinetic studies of anti-*H. pylori* regimens. These include high-performance liquid chromatography (HPLC), liquid chromatography coupled with tandem mass spectrometry (LC-MS/MS), and diode-array detection (DAD), among others [15,16,17,18,19,20,21,22,23]. However, many of these reported methods focus on older regimens involving PPIs and are not suitable for the concurrent quantification of vonoprazan along with traditional antibiotics. Furthermore, existing methods often involve laborious and time-consuming sample preparation procedures, such as solid-phase extraction which may hinder routine clinical application [24,25,26]. This creates a pressing need for the development of a more efficient, accurate, and robust analytical method that can simultaneously determine the three active components of Voquezna Triple Pak^®^ in human plasma.

Accurate quantification of vonoprazan, amoxicillin, and clarithromycin in biological matrices is essential for a variety of clinical and research purposes. These include pharmacokinetic and bioequivalence studies, therapeutic drug monitoring, dose optimization, and the evaluation of patient adherence. Among available techniques, LC-MS/MS has emerged as the gold standard in bioanalysis, owing to its unmatched selectivity, sensitivity, and suitability for multiplexed analysis in complex biological samples such as plasma [27,28]. LC-MS/MS enables simultaneous quantification of multiple analytes with high precision and minimal interference from endogenous components, making it ideal for clinical applications.

Despite the growing clinical relevance of the new vonoprazan-based triple therapy, a comprehensive literature review revealed a significant gap: no fully validated LC-MS/MS method has yet been reported for the simultaneous determination of vonoprazan, amoxicillin, and clarithromycin in human plasma. Addressing this gap is of great importance to support clinical development, pharmacokinetic profiling, and therapeutic monitoring of this recently approved regimen.

Therefore, the objective of the current study is to develop and validate a sensitive, specific, and reproducible LC-MS/MS method for the simultaneous quantification of vonoprazan, amoxicillin, and clarithromycin in spiked human plasma. A simple and efficient liquid–liquid extraction technique was employed for sample preparation, ensuring practicality and cost-effectiveness. The validated method can be readily applied in clinical and pharmacokinetic studies involving the novel Voquezna Triple Pak^®^, facilitating better understanding and the optimization of this promising anti-*H. pylori* therapy.

## 2. Results and Discussion

Method development began with a comprehensive literature review, which revealed a clear gap in the availability of analytical methods for the simultaneous determination of vonoprazan fumarate, amoxicillin, and clarithromycin in human plasma. While previous studies addressed the quantification of individual drugs or binary mixtures using various chromatographic techniques, no validated LC-MS/MS method has been reported for the concurrent analysis of this triple therapy regimen in plasma [29]. This gap highlights the novelty and clinical relevance of the present study. The developed LC-MS/MS method was compared with previously reported analytical methods for vonoprazan, amoxicillin, and clarithromycin, highlighting its novelty and superior performance. While most earlier studies focused on single or binary combinations, such as vonoprazan with its metabolites [17] or amoxicillin and clarithromycin [19,20], or applied HPLC methods to dosage forms and simulated fluids [15,18,22], no validated LC-MS/MS assay for the simultaneous determination of this ternary combination in human plasma has been reported. The proposed method employs liquid–liquid extraction using xylene, providing high and consistent recoveries (93–104%) with negligible matrix effects, unlike some previous approaches that suffered from moderate recoveries or interference [15,19,23]. Additionally, the method demonstrated excellent sensitivity with LLOQs of 2–5 ng/mL (0.004–0.01 ng/injection) and LODs of 0.0013–0.0033 ng/injection, substantially lower than those of reported HPLC [15,18] and electrochemical methods [16]. The short chromatographic run time of 5 min enhances throughput compared to earlier methods requiring 10–25 min per injection [15,18,22]. Furthermore, greenness assessment using the AGREE metric indicated superior environmental performance due to minimal solvent consumption and waste generation [18,21,23]. Overall, the method provides a fast, sensitive, selective, and greener alternative suitable for pharmacokinetic studies, bioequivalence evaluations, and therapeutic drug monitoring of vonoprazan-based triple therapy. The main objective was to establish a rapid, sensitive, and selective LC-MS/MS method that could effectively separate and quantify vonoprazan fumarate, amoxicillin, and clarithromycin.

### 2.1. Chromatographic Conditions

**Figure 1:**
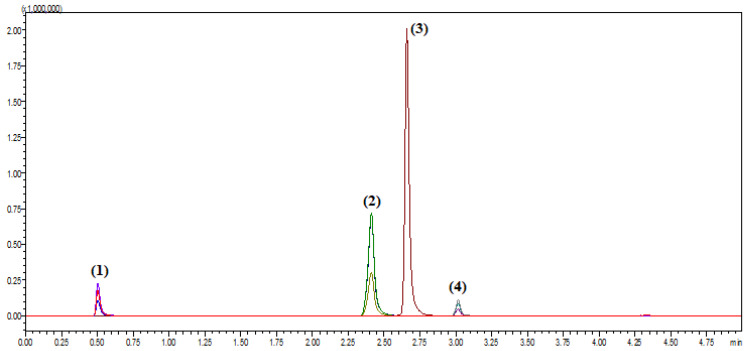
Total ion chromatogram (TIC) chromatograms for Amoxicillin (1), Vonoprazan Fumarate (2), Clarithromycin (3), and Diazepam (4) at 10 ng/mL.

In the initial stages of method development, different stationary phases such as C8, phenyl, and polar-embedded columns were tested. However, these columns did not achieve satisfactory resolution or acceptable peak shapes for all analytes. Ultimately, the Phenomenex Kinetex C18 column (50 × 3 mm, 2.6 µm) was selected based on its superior performance in terms of retention, peak symmetry, and resolution for the targeted drugs [30]. Attention was then directed to optimizing the mobile phase composition. Several combinations of aqueous and organic solvents with and without modifiers were evaluated. The best separation was achieved using a gradient elution with mobile phase A consisting of 0.1% formic acid in water and mobile phase B of 0.1% formic acid in acetonitrile. The gradient program started with 5% B for 0.5 min, increased linearly to 65% B by 1.5 min, held at 65% until 4.0 min, then returned to 5% B by 4.2 min. This program facilitated the elution of all analytes within a total run time of 5.0 min without compromising resolution, as shown in Figure 1. In parallel, the effect of mobile phase pH was examined, given its critical role in influencing analyte ionization and chromatographic behavior. Trials were conducted at various pH levels to improve ionization efficiency and peak sharpness. The incorporation of 0.1% formic acid into both aqueous and organic mobile phase components provided a stable acidic environment that enhanced analyte protonation, thereby improving detection in positive ion mode [31,32]. System suitability parameters are shown in Table 1.

### 2.2. Mass Spectrometric Conditions

To the best of our knowledge, no LC-MS/MS method has been reported for the simultaneous determination of vonoprazan, amoxicillin, and clarithromycin in human plasma. Therefore, the present study represents the first validated LC-MS/MS method for this ternary combination, employing diazepam as an internal standard. The superior sensitivity and selectivity of tandem mass spectrometry make it the preferred technique for the quantification of low-concentration analytes and their metabolites in complex biological matrices.

**Figure 2:**
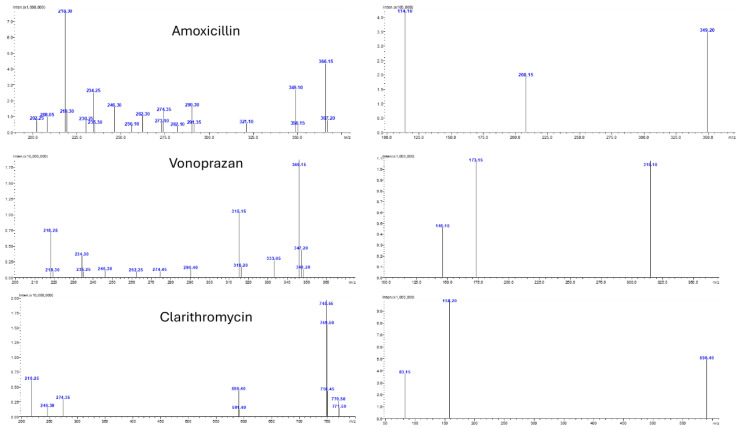
MS/MS Spectra of the analyzed drugs.

Mass spectrometric analysis was carried out using a Shimadzu LCMS-8050 triple quadrupole mass spectrometer equipped with an electrospray ionization (ESI) source operating in positive ion mode. The instrument was set to operate in Multiple Reaction Monitoring (MRM) mode to maximize specificity and sensitivity. The most intense and selective precursor-to-product ion transitions were carefully selected for each analyte and the internal standard. The optimized MRM transitions were m/z 366.05 → 114.10 for amoxicillin, m/z 346.05 → 315.10 for vonoprazan, m/z 748.30 → 158.20 for clarithromycin, and m/z 285.25 → 193.10 for diazepam as shown in Figure 2.

Instrumental parameters were optimized to improve ionization efficiency and signal stability. The nebulizer gas flow was set at 3.0 L/min, while both the drying gas and heating gas flows were maintained at 10.0 L/min. The interface temperature was adjusted to 300 °C, with the desolvation line (DL) and heating block temperatures set at 250 °C and 400 °C, respectively. A dwell time of 8 milliseconds was applied to each MRM transition to ensure reliable detection and quantification.

These optimized mass spectrometric conditions enabled the accurate and reproducible determination of vonoprazan, amoxicillin, and clarithromycin in spiked human plasma samples. Sharp, well-resolved peaks and excellent signal-to-noise ratios were obtained without significant interference from endogenous plasma components, supporting the method’s robustness for future pharmacokinetic or bioequivalence applications as shown in Figure 1.

### 2.3. Sample Preparation and Liquid–Liquid Extraction (LLE)

Efficient sample preparation plays a pivotal role in the accuracy, sensitivity, and reproducibility of bioanalytical methods, especially when working with complex matrices such as human plasma. In the early stages of method development, protein precipitation and solid-phase extraction (SPE) were considered, but both approaches gave suboptimal results; protein precipitation led to high matrix effects due to incomplete removal of plasma proteins and phospholipids, while SPE, although cleaner, was time-consuming and produced relatively lower and inconsistent recoveries. Consequently, liquid–liquid extraction (LLE) was selected as the preferred strategy because of its simplicity, efficiency, and cost-effectiveness, and it has long been recognized for effectively isolating analytes from biological matrices with minimal interference. To enhance extraction efficiency for the structurally diverse analytes, amoxicillin, vonoprazan, and clarithromycin, xylene was used as the organic solvent, as it offers good partitioning for moderately lipophilic compounds and has been previously applied in LLE protocols for macrolide antibiotics and weakly basic drugs [33,34]. Amoxicillin, which possesses both a primary amine (pKa ~7.4) and a carboxylic acid, predominantly exists as a zwitterion at the extraction pH; this zwitterionic character, while neutral in net charge, retains high polarity, limiting complete partitioning into the organic phase and affecting ionization efficiency in ESI [35]. In contrast, vonoprazan and clarithromycin, being more lipophilic and basic, exhibit higher MS/MS responses. The developed LLE protocol with xylene provided excellent recoveries, minimal matrix interferences, and clean chromatographic baselines, aligning with previous studies supporting the robustness of LLE for multicomponent drug analysis in biological fluids [36,37,38,39,40].

### 2.4. Method Validation

The developed method was validated in accordance with FDA guidelines, assessing parameters such as selectivity, linearity, precision, accuracy, sensitivity, recovery, and matrix effect using spiked human plasma samples [25].

### 2.5. Selectivity

**Figure 3:**
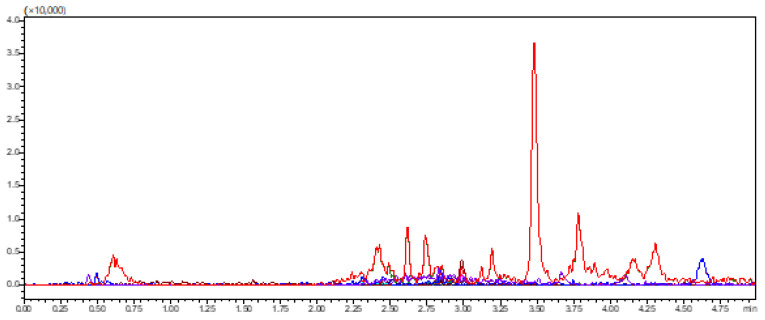
TICs of blank human plasma samples from two different sources, showing no interfering peaks at the retention times of the analytes.

**Figure 4:**
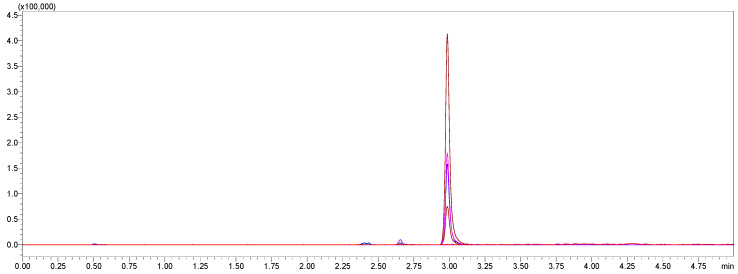
MRM chromatograms showing the internal standard (diazepam) peak at its retention time in blank plasma samples spiked.

Selectivity was confirmed by analyzing blank human plasma samples obtained from six different sources to ensure the absence of endogenous interference at the retention times of amoxicillin, vonoprazan fumarate, clarithromycin, and the internal standard, diazepam. Additionally, blank plasma samples spiked only with the internal standard were analyzed to verify the absence of interference with the analyte peaks. Representative chromatograms are presented in Figure 3 and Figure 4. Figure 3 illustrates the total ion chromatograms of blank plasma from two different donors (red and blue), confirming the absence of interfering peaks at the analytes’ retention times. Figure 4 demonstrates the multiple reaction monitoring (MRM) chromatograms of diazepam (internal standard) spiked at different concentrations, confirming the absence of interference with the analyte peaks.

### 2.6. Linearity and Range

The linearity of the developed LC-MS/MS method was evaluated by analyzing plasma samples spiked with known concentrations of the analytes within their respective calibration ranges. For amoxicillin and clarithromycin, the concentration range was 2–100 ng/mL, while for vonoprazan it was 5–100 ng/mL. Each calibration level was analyzed in triplicate. Calibration curves were constructed by plotting the peak area ratio of the analyte to the internal standard (IS) versus the concentrations ratios. Excellent linearity was observed for all analytes across the studied ranges, with correlation coefficients (r) exceeding 0.998. The regression equations were as follows: amoxicillin, Y = 0.1672X − 0.0439 (r = 0.9998); clarithromycin, Y = 3.3423X − 0.3026 (r = 0.9999); and vonoprazan, Y = 0.7858X − 0.4115 (r = 0.9983). These results confirm the method’s suitability for reliable quantification of the analytes within the specified ranges.

### 2.7. Accuracy and Precision

Accuracy was assessed by analyzing spiked plasma samples at four concentration levels: lower limit (LLQC), low (LQC), medium (MQC), and high (HQC). The calculated concentrations were compared to the nominal concentrations, and the percentage recovery was determined.

### 2.8. Accuracy

Precision was evaluated at the same QC levels by analyzing FIVE replicate samples. Within-run precision was assessed by measuring replicates on the same day, while between-run precision was determined by analyzing replicates across three consecutive days under identical experimental conditions. Precision results were expressed as the relative standard deviation (RSD%). Data of accuracy and precision are presented in Table 2.

### 2.9. Sensitivity

The developed LC-MS/MS method exhibited excellent sensitivity, as demonstrated by the successful quantification of the lower limit of quantification (LLOQ) for each analyte with acceptable performance. The LLOQ was defined as the lowest concentration on the calibration curve that could be quantified reliably, with both accuracy and precision meeting regulatory acceptance criteria. For each analyte, five replicate measurements at the LLOQ level confirmed that the signal response was at least five-fold higher than that of the zero calibrator, with accuracy within ±20% of the nominal value and precision (CV) not exceeding 20%. Using a 2 µL injection volume, the LLOQ corresponds to 0.004 ng/injection for amoxicillin, 0.004 ng/injection for clarithromycin, and 0.01 ng/injection for vonoprazan. The limits of detection (LOD), estimated based on a signal-to-noise ratio of 3:1, were approximately 0.0013 ng/injection for amoxicillin, 0.0013 ng/injection for clarithromycin, and 0.0033 ng/injection for vonoprazan. These results confirm the method’s capability to detect and quantify low plasma concentrations of amoxicillin, clarithromycin, and vonoprazan with high confidence.

### 2.10. Recovery and Matrix Effect

The extraction efficiency of the method was consistent and reproducible across all QC levels. Recovery was evaluated by comparing the peak responses of analytes in pre-extraction spiked plasma samples to those in post-extraction spiked samples at low (LQC), medium (MQC), and high (HQC) concentrations. The results demonstrated efficient and consistent recovery, indicating that the extraction procedure was robust and did not result in significant analyte loss.

Matrix effects were assessed to determine any potential ion suppression or enhancement caused by endogenous plasma components. This was done by comparing the responses of post-extraction spiked samples with those of equivalent concentrations prepared directly in the mobile phase. The minimal variation observed confirmed that matrix effects were negligible and did not interfere with analyte quantification. These findings, summarized in Table 3, support the method’s reliability and suitability for routine bioanalysis. Full detailed results are shown in Supplementary File Table S1.

### 2.11. Stability

To ensure the reliability of the method during routine bioanalytical applications, the stability of vonoprazan, amoxicillin, and clarithromycin in human plasma was thoroughly examined under a range of conditions simulating sample handling and storage scenarios. The evaluation included short-term room temperature exposure, long-term storage at −80 °C, three freeze–thaw cycles, benchtop handling, and autosampler storage following extraction. For each condition, stability was assessed using quality control samples at both low and high concentration levels.

Across all stability tests, the analyte concentrations remained within ±15% of their respective nominal values, with relative standard deviations not exceeding 15%, thereby meeting the acceptance criteria set by the FDA. These results, summarized in Table 4, confirm that the analytes are chemically stable under the tested conditions, reinforcing the suitability of the developed LC-MS/MS method for reliable use in clinical and pharmacokinetic studies. Full detailed results are shown in Supplementary File Table S2.

The validated method is suitable for pharmacokinetic and bioequivalence studies involving vonoprazan-based triple therapy. The co-administration of vonoprazan fumarate with amoxicillin and clarithromycin for the treatment of *H. pylori* has recently received FDA approval, highlighting the need for a selective, sensitive, and robust bioanalytical method for therapeutic drug monitoring [41].

### 2.12. Analytical GREEnness Metric (AGREE)

In June 2020, a new technique was introduced involving a downloadable greenness assessment software named AGREE, which is based on the twelve fundamental principles of Green Analytical Chemistry (GAC). In AGREE, the final greenness score is expressed as a fraction between zero and one, where values closer to one indicate a greener method, while values near zero reflect poor greenness. The software generates a circular pictogram divided into twelve segments, each representing one of the GAC principles. These segments are color-coded from green to red and automatically adjusted in width according to their relevance. The overall score is displayed at the center of the pictogram [42,43].

**Figure 5:**
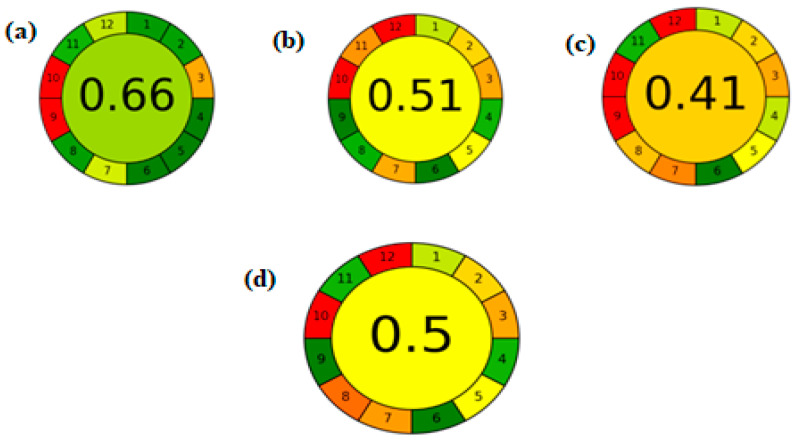
AGREE assessment tool comparison between the proposed method (**a**) and the reported methods from references [18,20,23], represented in (**b**–**d**), respectively. The AGREE pictograms visually reflect the compliance of each method with the twelve principles of green analytical chemistry, with scores closer to 1 indicating higher greenness.

The AGREE tool was applied to assess the greenness of the developed method, as illustrated in Figure 5. The evaluation revealed that the proposed method is greener than previously reported methods. However, it did not achieve an excellent greenness score due to the high energy consumption associated with the use of tandem mass spectrometry. Nonetheless, the method still demonstrates superior greenness compared to existing approaches.

## 3. Materials and Methods

### 3.1. Materials and Reagents

**Figure 6:**
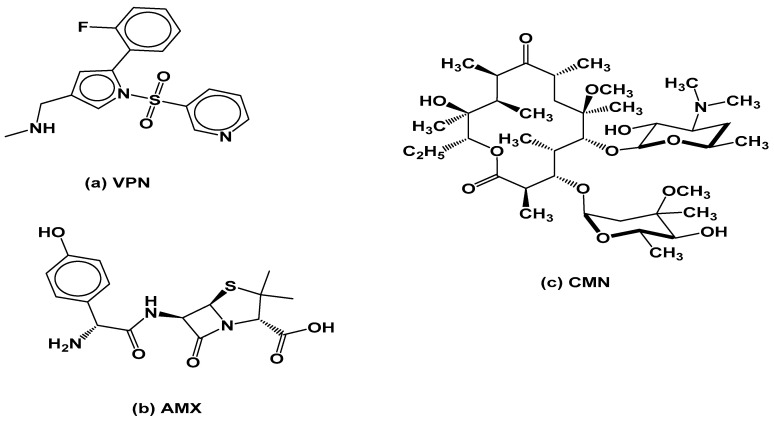
Chemical structures of (**a**) Vonoprazan (VPN), (**b**) Amoxicillin (AMX), (**c**) Clarithromycin (CMN).

Vonoprazan fumarate, Amoxicillin trihydrate, Clarithromycin, and Diazepam (used as the internal standard) were purchased from Sigma-Aldrich (St. Louis, MO, USA). The purity of all reference standards was certified to be ≥98.5%. The chemical structures of the studied compounds are presented in Figure 6.

Commercial triple therapy tablets (Voquezna^®^ Triple Pak), each containing Vonoprazan (20 mg), Amoxicillin (500 mg), and Clarithromycin (500 mg), were used for the assay and kindly provided by Egyptian Pharmaceutical Company (Cairo, Egypt).

Acetonitrile and formic acid of LC-MS grade were obtained from Sigma-Aldrich (Darmstadt, Germany).

Deionized water was prepared in-house using a Milli-Q purification system (Millipore, Bedford, MA, USA) and is referred to as “water” throughout the manuscript.

Human blank plasma was supplied by VACSERA (The Holding Company for Biological Products and Vaccines, Cairo, Egypt) and stored at −80 °C until use.

### 3.2. Instruments

The chromatographic separation was performed using a Shimadzu UHPLC-MS/MS system consisting of a Nexera-i LC-2040C UHPLC (Shimadzu, Kyoto, Japan) coupled with an LCMS-8050 triple quadrupole mass spectrometer equipped with an electrospray ionization (ESI) source. System control and data acquisition were conducted using LabSolutions software version 6.86 (Shimadzu, Kyoto, Japan).

Separation was carried out on a Phenomenex Kinetex C18 column (50 mm × 3 mm, 2.6 μm particle size, Phenomenex, Torrance, CA, USA) maintained at 40 °C.

### 3.3. Procedures

#### 3.3.1. Preparation of Standard Sample Solutions

Standard stock solutions of amoxicillin, vonoprazan fumarate, and clarithromycin were prepared at 1 mg/mL in a 50:50 (*v*/*v*) mixture of acetonitrile and water. Diazepam, used as the internal standard (IS), was prepared at 100 μg/mL in the same solvent. These stock solutions were subsequently diluted to working standard solutions at concentrations of 100 μg/mL for the analytes and 10 μg/mL for the IS.

In accordance with FDA bioanalytical method validation guidelines, calibration standards and reference solutions were prepared by spiking into the same biological matrix as the test samples; human plasma [25].

For plasma spiking, amoxicillin and clarithromycin calibration standards were prepared at concentrations of 2, 5, 10, 20, 50, and 100 ng/mL, while vonoprazan standards were prepared at 5, 10, 20, 30, 50, and 100 ng/mL. Diazepam was added to all samples at a fixed concentration of 4 ng/mL.

Quality control (QC) samples in plasma were independently prepared at four concentration levels, lower limit (LLQC), low (LQC), medium (MQC), and high (HQC), corresponding to 2, 6, 50, and 100 ng/mL for amoxicillin and clarithromycin, and 5, 15, 30, and 100 ng/mL for vonoprazan, respectively.

All stock and working solutions were freshly prepared and stored at −80 °C to maintain analyte stability. Given the reported instability of amoxicillin and vonoprazan fumarate at low concentrations [44], working solutions were prepared immediately before use to minimize degradation.

#### 3.3.2. Sample Preparation for LC-MS/MS

Plasma samples were stored at −80 °C until analysis and thawed immediately before use. Extraction of amoxicillin, vonoprazan fumarate, clarithromycin, and the internal standard (diazepam) was performed using liquid–liquid extraction (LLE).

An aliquot of 200 μL of spiked plasma sample was transferred into clean tubes, followed by the addition of 300 μL of acetonitrile. The mixture was vortexed thoroughly for complete homogenization. Subsequently, 5 mL of xylene was added, and the mixture was alkalinized by adding sodium hydroxide solution. The sample was vortexed again for one minute using high shear mixing to facilitate efficient extraction.

The suspension was centrifuged at 10,000 rpm for 10 min to promote phase separation. The upper organic layer was carefully collected and evaporated to dryness under a gentle stream of nitrogen at 45 °C. The resulting dry residue was reconstituted in 100 μL of the initial mobile phase composition (5% acetonitrile and 95% aqueous phase) and transferred to autosampler vials for subsequent LC-MS/MS analysis.

#### 3.3.3. Chromatographic Conditions

Chromatographic separation was performed on a Phenomenex Kinetex C18 column (50 × 3 mm, 2.6 µm particle size) maintained at 40 °C. The mobile phase consisted of Phase A (0.1% formic acid in water) and Phase B (0.1% formic acid in acetonitrile).

A gradient elution program was employed at a flow rate of 0.50 mL/min. The gradient started with 5% Phase B held for 0.5 min, followed by a linear increase to 65% Phase B at 1.5 min. This composition was maintained until 4.0 min, then rapidly decreased back to 5% Phase B at 4.2 min. The total run time was 5.0 min, after which the column was equilibrated at 5% Phase B for the next injection. The injection volume was 2 µL.

#### 3.3.4. Mass Spectrometry Conditions

Mass spectrometric analysis was performed using a Shimadzu LCMS-8050 triple quadrupole mass spectrometer (Shimadzu, Kyoto, Japan) equipped with an electrospray ionization (ESI) source. Ionization was carried out in positive mode under the following source parameters: nebulizer gas flow rate of 3.0 L/min, drying gas flow rate of 10.0 L/min, and heating gas flow rate of 10.0 L/min. The interface temperature was maintained at 300 °C, the desolvation line (DL) temperature at 250 °C, and the heating block temperature at 400 °C to ensure optimal ionization efficiency and analyte stability.

The instrument operated in multiple reaction monitoring (MRM) mode to provide high sensitivity and specificity for target analytes. The dwell time for each MRM transition was set to 8 ms. Detailed MRM transitions, including precursor ions, product ions, and optimized collision energies (CE), are summarized in Table 5. Quantification of each compound was based on the most intense product ion signal.

### 3.4. Data Acquisition and Analysis

Data acquisition was performed using LabSolutions software version 6.86. Calibration curves were generated from standard solutions, and the concentrations of the analytes in the samples were quantified from the regression equations of the calibration curves.

### 3.5. Validation

The bioanalytical method was validated according to FDA guidelines to assess selectivity, linearity, precision, accuracy, sensitivity, recovery, and matrix effects using spiked human plasma samples [25].

### 3.6. Linearity and Range

Linearity was evaluated by constructing calibration curves in spiked human plasma across the expected quantitation range for each analyte. In accordance with FDA recommendations, each analytical run included a complete calibration set consisting of a blank plasma sample (without analytes or internal standard), a zero calibrator (blank plasma spiked with the internal standard only), and six non-zero calibrators covering the entire quantitation range, including the lower limit of quantification (LLOQ). The non-zero concentrations for amoxicillin and clarithromycin were 2, 5, 10, 20, 50, and 100 ng/mL, while those for vonoprazan were 5, 10, 20, 30, 50, and 100 ng/mL. Diazepam was added as the internal standard at a fixed concentration of 4 ng/mL in all calibration samples.

After liquid–liquid extraction, the samples were injected into the LC-MS/MS system. Calibration curves were constructed by plotting the peak area ratios against concentration ratios. Each calibration level was injected in triplicate from the same batch.

### 3.7. Accuracy and Precision

Accuracy and precision were evaluated by analyzing plasma samples spiked at fourquality control (QC) levels, lower limit (LLQC), low (LQC), medium (MQC), and high (HQC), corresponding to 2, 6, 50, and 100 ng/mL for amoxicillin and clarithromycin, and 5, 15, 30, and 100 ng/mL for vonoprazan, respectively. Each QC level was analyzed in five replicates. Accuracy was assessed by comparing the measured concentrations to the nominal values and expressed as the percentage recovery. Precision was assessed as both within-run (intra-day) and between-run (inter-day) precision. Within-run precision was determined by analyzing five replicates of each QC level within a single day, while between-run precision was evaluated by analyzing five replicates of each QC level over three consecutive days under the same experimental conditions. Precision results were expressed as the relative standard deviation (RSD%).

### 3.8. Sensitivity

The sensitivity of the developed LC-MS/MS method was determined by establishing the lower limit of quantification (LLOQ), defined as the lowest non-zero standard on the calibration curve that could be quantified with acceptable accuracy and precision. The accuracy and precision at the LLOQ were evaluated using five replicates.

### 3.9. Recovery and Matrix Effect

Recovery was calculated by comparing peak areas from extracted spiked plasma samples with those from post-extraction spiked plasma at LQC, MQC, and HQC levels. Matrix effect was assessed by comparing peak areas of post-extraction spiked plasma samples to those of pure standard solutions prepared in mobile phase.

### 3.10. Selectivity

Selectivity was confirmed by analyzing blank plasma samples from six different sources to ensure the absence of interfering peaks at the retention times of amoxicillin, vonoprazan fumarate, clarithromycin, and the internal standard diazepam.

### 3.11. Stability

Stability of vonoprazan fumarate, amoxicillin, and clarithromycin in human plasma was evaluated under various conditions to ensure the reliability of the analytical method during sample handling, storage, and analysis. The following stability assessments were conducted in accordance with FDA bioanalytical method validation guidelines [25]:

1. Short-Term Stability:

QC samples at low and high concentration levels were kept at room temperature (25 °C) for 6 h to simulate the conditions during routine sample processing. Samples were then analyzed and compared to freshly prepared samples.

2. Long-Term Stability:

To assess long-term storage stability, QC samples were stored at −80 °C for a minimum of 30 days. At the end of the storage period, samples were analyzed and concentrations were compared with time-zero values.

3. Freeze–Thaw Stability:

Samples were subjected to three freeze–thaw cycles by freezing at −80 °C for 24 h and thawing at room temperature. After the third cycle, the samples were analyzed and results were compared with freshly prepared QC samples.

4. Benchtop Stability (Room Temperature Stability):

QC samples were left at room temperature (approximately 25 °C) for 12 h to simulate sample handling during preparation and analysis. Concentrations were then determined and compared with nominal values.

5. Post-Preparative Stability (Autosampler Stability):

Processed QC samples were stored in the autosampler at 10 °C for 24 h before reinjection to assess stability after preparation. The results were compared to initial values obtained from freshly injected samples.

## 4. Conclusions

In this study, a robust, rapid, and highly sensitive LC-MS/MS method was successfully developed and validated for the simultaneous quantification of vonoprazan, amoxicillin, and clarithromycin in spiked human plasma. This method addresses a critical gap in bioanalytical techniques by enabling the concurrent analysis of all three components of the newly FDA-approved Voquezna^®^ Triple Pak—currently considered a promising therapy for *Helicobacter pylori* eradication. The simplicity of the sample preparation using liquid–liquid extraction, combined with a short chromatographic run time of 5 min, enhances the method’s practicality and throughput for routine clinical and pharmacokinetic applications.

The method demonstrated excellent linearity for each analyte over the studied concentration ranges with correlation coefficients exceeding 0.999, indicating high accuracy and reliability. It also showed satisfactory recovery, precision, and minimal matrix effect, with all validation parameters complying with FDA bioanalytical method guidelines. The optimized conditions, including the use of a Phenomenex Kinetex C18 column and gradient elution with 0.1% formic acid in water and acetonitrile, ensured high chromatographic efficiency and reproducibility.

This validated LC-MS/MS assay offers significant advantages over previously reported methods by being faster, simpler, and more sensitive, especially for the newly introduced vonoprazan. Its applicability in pharmacokinetic studies, therapeutic drug monitoring, and bioequivalence evaluations makes it a valuable tool for supporting clinical use and further research on vonoprazan-based therapies. This method is expected to contribute to improved patient management and therapy optimization in the treatment of *H. pylori* infections.

## Figures and Tables

**Table 1:** System suitability parameters for the analysis of the compounds studied.

Compound	tR(min)	k′	N	HETP (µm)	Resolution (Rs)	Selectivity (α)
Amoxicillin	0.5	4	400	125	14.4	5.5
Vonoprazan	2.3	22	3762	13.3	2.7	1.18
Clarithromycin	2.7	26	5184	9.65	2.4	1.12
Diazepam (IS)	3	29	14,400	3.47	—	—

**Table 2:** Accuracy and precision data for determination of the drugs in spiked human plasma samples.

QC Level	Nominal Conc. (ng/mL)	Amoxicillin Recovery % *	Within-Run RSD % *	Between-Run RSD % *	Clarithromycin Recovery % *	Within-Run RSD % *	Between-Run RSD %*	Vonoprazan Recovery % *	Within-Run RSD % *	Between-Run RSD % *
LLQC	2 (Amox & Clarith)/5 (Vonop)	112.46	5.1	6.4	99.56	1.2	2.1	114.89	1.7	7.4
LQC	6 (Amox & Clarith)/15 (Vonop)	105.58	3.4	2.5	101.26	2.5	3.1	106.48	4.2	2.8
MQC	10	99.2	1.7	2.1	99.46	0.4	2.1	103.69	2.2	6
HQC	100	100.4	3.9	5.9	99.91	1.5	2.2	101.36	4.6	3.1

* The mean value should be within 15% except at LLOQ, where it should be within 20%.

**Table 3:** Extraction efficiency and matrix effects for determination of the three drugs.

QC Level	Nominal Conc. (ng/mL)	Amoxicillin Extraction Efficiency (%)	Amoxicillin Matrix Effect (%)	Clarithromycin Extraction Efficiency (%)	Clarithromycin Matrix Effect (%)	Vonoprazan Extraction Efficiency (%)	Vonoprazan Matrix Effect (%)
LQC	2 (Amox & Clarith)/5 (Vonop)	93.3	111.2	96.6	113.0	109.3	103.6
MQC	10	101.8	95.6	97.0	97.5	104.1	100.0
HQC	100	93.4	101.6	98.3	101.2	102.7	99.8

**Table 4:** Stability data for the studied drugs in human plasma.

Stability Parameter	Vonoprazan Fumarate		Amoxicillin		Clarithromycin	
	5 ng/mL	100 ng/mL	2 ng/mL	100 ng/mL	2 ng/mL	100 ng/mL
Short-term stability	98.7	101.6	100.5	100.9	100.0	93.2
Long-term stability	105.5	98.7	96.1	97.3	93.7	88.7
Freeze–thaw stability	90.1	102.4	97.7	97.1	101.8	95.5
Benchtop stability	107.3	101.7	96.4	101.1	93.2	96.1
Autosampler stability	99.2	95.9	101.4	94.3	100.0	93.2

**Table 5:** MRM optimized parameters for the analysis of the studied drugs.

Compound Name	Precursor Ion	Product Ion	CE (V)
Amoxicillin	366.05	114.10 *	−22
		349.20	−11
		208.15	−14
Vonoprazan Fumarate	346.05	173.15	−27
		315.10 *	−13
		146.15	−46
Clarithromycin	748.30	158.20 *	−30
		590.40	−21
		83.15	−50
Diazepam	285.25	193.10 *	−32
		154.10	−27
		222.10	−26

* Ion was used for quantification.

## Data Availability

The original contributions presented in this study are included in the article. Further inquiries can be directed to the corresponding author.

## Supplementary Materials

The following supporting information can be downloaded at: https://www.mdpi.com/article/10.3390/ph18101509/s1, Table S1: Internal Standard normalized Matrix Effect (ME), and extraction recovery (ER) in human drug-free plasma. (n = 6 for each level of QC samples). Table S2: Stability of AMX, VPN and CMN under various storage conditions (n =3 for each analyte concentration).
