# Supplementary material for: LC-MS/MS Method Development and Validation for Clinical Pharmacokinetics and Therapeutic Drug Monitoring of Potassium-Competitive Acid Blocker Vonoprazan-Based Triple Therapy for H. pylori in Human Plasma
Source: Pharmaceuticals (Basel). 2025 Oct 8;18(10):1509. doi: 10.3390/ph18101509 (PMC12567155; doi:10.3390/ph18101509)
Supplement: Supplementary file 1 [file pharmaceuticals-18-01509-s001.zip › pharmaceuticals-3812487-supplementary.pdf]

Table S1
Internal Standard normalized Matrix Effect (ME), and extraction recovery (ER) in human drug-free plasma. (n = 6 for each level of QC samples).

		Matrix effect (%)		Extraction recovery (%)	
Nominal concentration (ng/mL)	Level	Mean	CV	Mean	CV
**AMX**					
5	LQC	111.2	4.3	93.3	8.1
20.0	MQC	95.6	3.3	101.8	2.1
100.0	HQC	101.6	2.6	93.4	3.2
**VPN**					
5	LQC	103.6	6.9	109.3	7.3
20.0	MQC	100.0	2.8	104.1	2.8
100.0	HQC	99.8	3.7	102.7	3.0
**CMN**					
5	LQC	113.0	5.8	96.6	3.4
20.0	MQC	97.5	3.1	97.0	2.4
100.0	HQC	101.2	2.4	98.3	1.7

CV. coefficient of variation. LQC, MQC and HQC3 correspond to the QC concentrations at low, medium and high level, respectively; AMX, VPN; and CMN.

Table S2
Stability of AMX, VPN and CMN under various storage conditions (n =3 for each analyte concentration).

	After three freeze-thaw cycles		Before extraction: RT (24 h)		Before extraction: 4°C (48 h)		Before extraction: -20°C (30 d)		Auto-sampler: 4 °C (24 h)	
Nominal concentration (ng/mL)	Bias[a] (%)	CV (%)	Bias[a] (%)	CV (%)	Bias[a] (%)	CV (%)	Bias[a] (%)	CV (%)	Bias[a] (%)	CV (%)
**AMX**										
5	96.7	1.8	96.4	5.9	100.5	4.8	96.1	9.2	101.4	10.3
20	88.9	6.5	94.7	5.3	99.8	8.4	97.9	4.3	95.7	5.7
100	97.1	3.2	101.1	10.2	100.9	6.3	97.3	2.9	94.3	5.2
**VPN**										
5	90.1	3.8	107.3	2.1	98.7	4.6	105.5	5.2	99.2	7.9
20	94.5	6.2	103.7	3.2	99.4	8.8	98.8	4.5	93.6	4.2
100	102.4	2.7	101.7	5.8	101.6	4.1	98.7	2.4	95.9	5.6
**CMN**										
5	101.8	3.4	93.2	9.9	100.0	7.3	93.7	2.4	101.8	3.4
20	98.2	2.3	93.9	3.3	97.3	1.7	88.0	4.3	98.2	2.9
100	95.5	2.8	96.1	1.5	93.2	3.1	88.7	2.1	95.5	3.3

CV. coefficient of variation; d. day; h. hour; RT. room temperature -
[a] Expressed as the mean percentage change from nominal concentration
